# The climate niche of the invasive tick species *Hyalomma marginatum* and *Hyalomma rufipes* (Ixodidae) with recommendations for modeling exercises

**DOI:** 10.1007/s10493-023-00778-3

**Published:** 2023-03-07

**Authors:** Agustín Estrada-Peña

**Affiliations:** 1 Department of Animal Health, Faculty of Veterinary Medicine, Miguel Servet 177, Zaragoza, 50013 Spain; 2 Group of Research on Emerging Zoonoses, Instituto Agroalimentario de Aragón (IA2), Zaragoza, Spain

**Keywords:** *Hyalomma marginatum*, *Hyalomma rufipes*, Climate niche, Air water contents

## Abstract

The finding of immature stages of some *Hyalomma* spp. feeding on migratory birds in Europe is unexceptional. The reports of adults of *Hyalomma* in Europe (incl. the British Isles) after successful molting from immatures have increased in recent years. It has been claimed that the warming of the target territory could favor the populations of these invasive ticks. Although evaluations of the impact on health or measures of adaptation are on their way, the climate niches of these species remain undefined, preventing preventive policies. This study delineates such niches for both *Hyalomma marginatum* (2,729 collection points) and *Hyalomma rufipes* (2,573 collections) in their distribution area, together with 11,669 points in Europe where *Hyalomma* spp. are believed to be absent in field surveys. Niche is defined from daily data of temperature, evapotranspiration, soil humidity and air saturation deficit (years 1970–2006). A set of eight variables (annual/seasonal accumulated temperature and vapor deficit) has the maximum discriminatory power separating the niches of both *Hyalomma* and a negative dataset, with an accuracy near 100%. The sites supporting *H. marginatum* or *H. rufipes* seem to be controlled by the joint action of the amount of water in the air (accounting for mortality) and the accumulated temperature (regulating development). The use of accumulated annual temperature as the only variable for predictive purposes of colonization of *Hyalomma* spp. looks unreliable, as far as values of water in air are excluded.

**Supplementary Information:**

The online version contains supplementary material available at 10.1007/s10493-023-00778-3.

## Introduction

In the last years, records of both *Hyalomma marginatum* and *Hyalomma rufipes* have become more common in central and northern Europe, not only as immatures detected on migratory birds (e.g., Hoffman et al. 2021) but also as adults feeding on large animals (e.g., Grandi et al. 2020). These two species seem to be showing a northward spread in Europe (Chitimia-Dobler et al. 2019). To note, that increase of records should not be directly correlated with a higher abundance of *Hyalomma* spp. in central and northern Europe, as we lack a baseline set of records, other than the pioneering efforts (Hoogstraal and Kaiser 1958; Hoogstraal et al. 1961, 1964). An increased awareness may be behind the recent increase of records of these ticks in northern latitudes, out of its ‘historical’ range. The topic is of importance as *Hyalomma* spp. are among the vectors of several pathogens affecting human health, most notably the etiological agent of the Crimean-Congo hemorrhagic fever (CCHF). Other than sporadic cases in Mediterranean countries (Negredo et al. 2019), an epidemic begun in Turkey around the year 2000 (Yilmaz et al. 2009). Although the causes of the origin of the epidemic are not yet clear (e.g., importation of infected ticks, mutation of strains of the virus, etc.) the incidence has slightly decreased, even if a country-wide eradication program is missing. The etiological agent of CCHF is a tick-borne virus that is maintained in nature in an enzootic vertebrate-tick-vertebrate cycle (Spengler and Estrada-Peña 2018). Ticks of the genus *Hyalomma* seem to be key in maintaining silent endemic foci. However, why they have a central role in the ecology of CCHF is unclear (Spengler and Estrada-Peña 2018). Concerns exist about the existence of suitable climate and host availability to cause an emerging epidemic by the virus in Europe (Gale et al. 2010; England 2015) or the existence of permanent populations of *Hyalomma* spp. (McGinley et al. 2021). Active surveys are being developed in several countries (e.g., De Liberato et al. 2018; Negredo et al. 2019; Sánchez-Seco et al. 2022), complemented by the efforts of citizen science (Foldvari et al. 2022).

The hypothesis of a deep freeze in winter as the main driver of population’s life cycle, was proposed by Hoogstraal (1979) citing soviet studies. This hypothesis was dominant among researchers until such concept of an absolute minimum threshold was replaced by an approach involving the accumulated temperature (i.e., the sum of degree days) allowing temperature-dependent processes to be completed for one generation before the mortality rate reaches 100% (Estrada-Peña et al. 2011, 2012). This would result in permanent populations of the tick. A site could hypothetically favor permanent populations of *Hyalomma* spp. if the sum of temperature (development) is above a species-specific threshold. Below such threshold, the development would be longer, and the mortality higher. Morel (1965) indicated that the northern limit of *H. marginatum* approximately overlaps the isotherm of 22 ºC in July. Maps by the World Health Organization (WHO) provide the presumed northern limit of *H. marginatum* as deduced from such limiting temperature that could theoretically preclude the completion of one generation before 100% of mortality (see https://cdn.who.int/media/docs/default-source/documents/health-topics/crimean-congo-haemorrhaigc-fever/introduction-to-crimean-congo-haemorrhagic-fever.pdf?sfvrsn=14c8c199_2&download=true).

Information about the range of temperature in which either *H. marginatum* or *H. rufipes* could prevail is fragmentary and has been obtained under laboratory conditions, as far as we know, without a careful evaluation of the mortality under a humidity range (Knight et al. 1978; Chen et al. 2012). The content of water in the air has been neglected in the definition of the weather niches of *Hyalomma* spp. Data about the life cycle of these ticks are deduced from observations of field activity at short intervals or while feeding on hosts without the capture of the long-term conditions under which they could prevail (e.g., Telmadarraiy et al. 2010; Hosseini-Chegeni et al. 2013; Choubdar et al. 2019). These field observations contain information of interest for the zone surveyed, indeed; however, its extrapolation and applicability to other areas is problematic. The current research on the topic prioritizes the reporting of features of the life cycle in incubators under constant conditions, as summarized by Valcárcel et al. (2020). Moreover, the amount of water in the air or the evaporation changes with the temperature – this behavior of water contents in the air according to temperature is commonly neglected in studies on the topic.

Two developments to model the life cycle of *H. marginatum* exist, namely a method driven by processes (i.e., equations that simulate development and mortality rates) proposed by Estrada-Peña et al. (2011), later adhered with improvements by England (2015); another one is based on statistical matches between climate features and the records of the tick in the Mediterranean region and Turkey (Estrada-Peña et al. 2007, 2012). As far as we know, similar models do not exist for *H. rufipes*, another tick carried by birds and introduced from Africa to Europe (Pascucci et al. 2019; Hoffman et al. 2020) but laboratory studies exist (Chen et al. 2012). Both approaches produced the same conclusion for *H. marginatum*: the tick needs a temperature sum of around 3,000–4,000 ºC to complete its life cycle in 1 year, disregarding the mortality. This has been assumed to be a pragmatic approach to capture the probability of completing the life cycle by imported ticks into central and northern Europe. However, the niche of a tick (actually, of every organism) is a hyper-dimensional volume (Soberón and Peterson 2020), commonly called the climate niche breadth (Chejanovski and Wiens 2014). Its over-simplification to a threshold of one single variable is unrealistic. Also, attempts to simplify such hyper volume (i.e., using a principal components reduction) result in the immediate loss of ecological meaning.

The main purpose of this study is to demonstrate that sites known to support permanent populations of either *H. marginatum* or *H. rufipes* can be characterized by the simultaneous use of several climate variables, instead of the calculation of the accumulated temperature alone. This study does not deal with crude range maps but aims to (i) provide an array of ecologically sound and statistically correct methods capturing the abiotic niche of two invasive ticks, *H. marginatum* and *H. rufipes*, of applicability to any other species of tick, (ii) build a dataset of climate data associated to *H. marginatum* and *H. rufipes* for further work on the topic, and (iii) to demonstrate the differences between the niches of these ticks compared against the climate in sites where they have been historically absent. Results further corroborate the usefulness of harmonic regression methods for obtaining daily estimates of climate values from online available climate datasets, delimiting the duration of seasons, obtaining evapotranspiration rates, and water pressure deficit, delineating the *dimensions* of the environmental niche of these ticks.

## Materials and methods

### Sources of records of *Hyalomma marginatum* and *H. rufipes*

Several sources of data were used for assembling the final dataset (Supplementary Material 1). Regarding *H. marginatum* the main source was a dataset with the published records of the tick in Europe and near territories already published by Estrada-Peña and de la Fuente (2016) and publicly available in Dryad (10.5061/dryad.2h3f2). Regarding *H. rufipes* a multi-source set of records was used, including (i) the original compilation of ticks by G.S. Cumming (1998) kindly provided by the author, (ii) the holdings of the collection of P.-C. Morel, kindly provided by Laurence Vial (INRA, Montpellier, France), (iii) data on the distribution of *H. rufipes* in South Africa, Namibia and Botswana, kindly provided by Ivan Horak (later published by Horak et al. 2018) and (iv) data about the distribution of *Hyalomma glabrum*, as published by Apanaskevich and Horak (2006); *H. glabrum* has been previously confused with *H. rufipes* and these data were used to remove erroneously identified records. This procedure resulted in 2,729 unique coordinates of *H. marginatum* and 2,573 of *H. rufipes*. The literally dozens of records of both species compiled by Morel (1965) were not included because they were collected while feeding on birds. Therefore, it is impossible to ascertain whether these were ‘traveling’ ticks on migratory birds or represent established local populations.

**Fig. 1:**
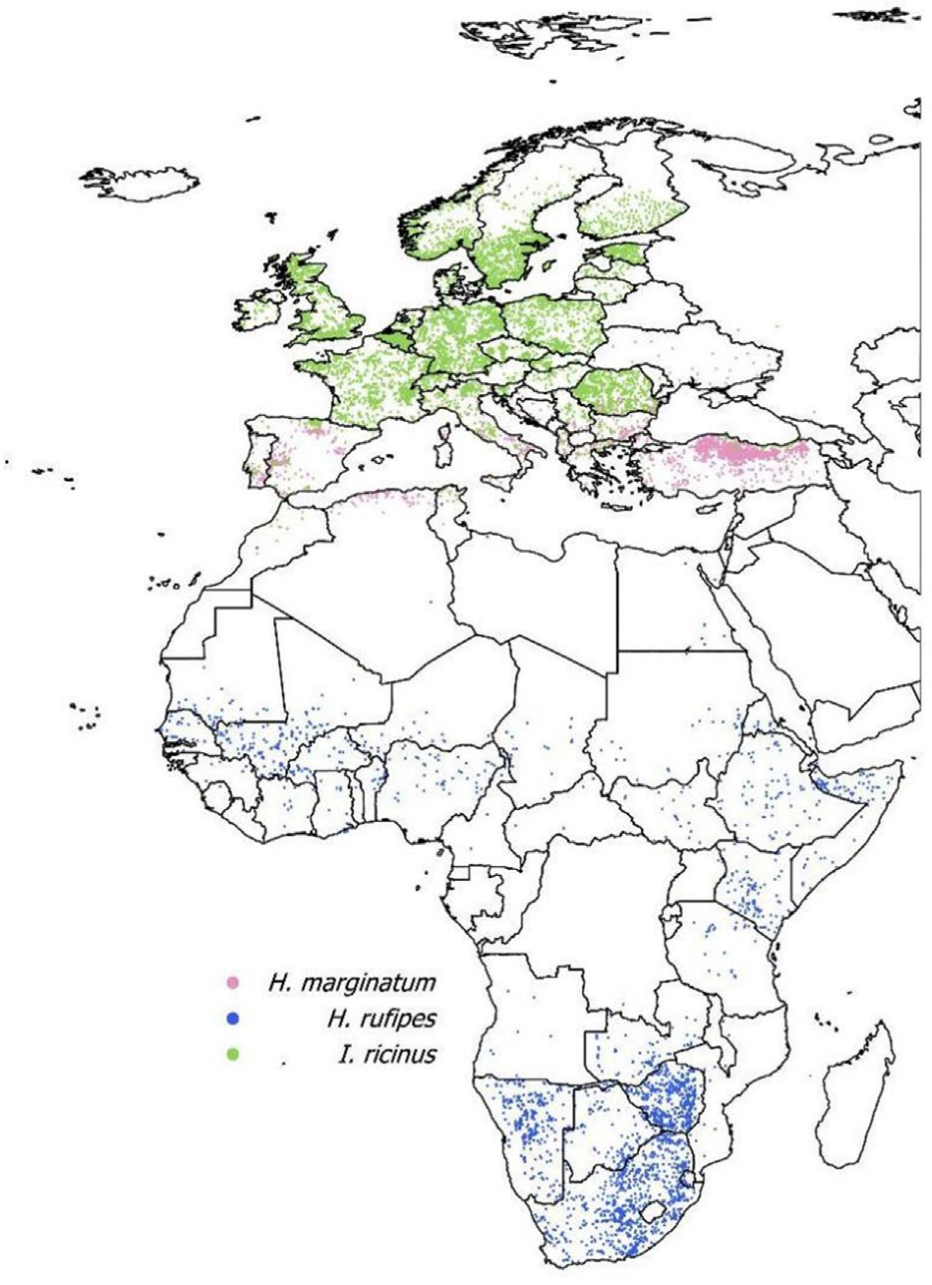
The geographical distribution of points used for this study. Both *Hyalomma marginatum* and *H. rufipes* are the positive set. The dataset of *Ixodes ricinus* was used as negative set for *Hyalomma*, to obtain a group of climate traits against which compare this of the positive dataset. The original data for the compilation of records are explained in ‘Material and Methods’

A set of negative sites in which the target ticks have not been recorded provide an objective comparison of the positive and negative niches. The selection involved the choice of a set of points representing the distribution of another tick species, since several criticisms have been raised to the algorithmic generation of pseudo-absences (Wisz and Guisan 2009; Barbet-Massim et al. 2012; Senay et al. 2013). The approach is based on the fact that the finding of other ticks in a site, together with the lack of reports of *Hyalomma* spp., most probably implies a true absence because collections in Europe would report *Hyalomma* spp. given its overall interest in the territory. Therefore, the records of *Ixodes ricinus* already published by Estrada-Peña and de la Fuente (2016) and publicly available in Dryad (10.5061/dryad.2h3f2) were used. The known distribution of *I. ricinus* represents a large portion of the European environment and provides a total of 11,669 points where *Hyalomma* spp. is considered absent. Figure 1 includes the spatial distribution of the three sets of points.

### Sources of climate data

Climate data from a public repository (http://www.climatologylab.org/terraclimate.html, last accessed June 2022) including monthly estimates of several variables dating back to 1958 were used. Records representing permanent populations of *H. marginatum* or *H. rufipes* were collected approximately in the period 1990–2006. However, a part of the records of *H. rufipes* were compiled from different sources (see above) and recorded before the year 1990. Therefore, climate data from the period extending between 1970 and 2006 were used.

Monthly data of maximum and minimum temperature (to obtain average values of temperature), soil humidity, actual evapotranspiration, and water vapor deficit in the air were downloaded and used for calculations. Each set of variables (e.g., soil humidity) was converted to monthly averages with the data for the period 1970–2006 to calculate one complete average year. These calculations were done with the R package *raster* (Hijmans 2022; R Core Team 2022). Instead of raw monthly variables, the coefficients of a harmonic regression of the monthly series of values of each variable were used to build descriptive climate features. The rationale is that a harmonic regression of monthly values at each pixel may reconstruct the daily series of climate values with an estimated error of about 0.5-4% in Europe (Estrada-Peña et al. 2014). The daily time series is the basis for subsequent calculations.

Fixed calendar dates (either meteorological or astronomical) are a human construct that cannot define the ecological meaning of seasons of the year. Daily values of average temperature along a natural year allowed to estimate the beginning and end of the meteorological seasons (i.e., spring, summer, etc.) detecting changes in the tendency of the daily time series of temperature, using the R package *changepoint* (Killick et al. 2022). It is possible to drawn the four annual seasons calculating four consecutive changes in the time series. To note, these seasons depend upon average temperature, and have a different length according to latitude and longitude. This method for calculating seasons may produce inconsistent results because the records of *H. rufipes* extend above and well below the equator in Africa. The seasons are inverted in both hemispheres. Further, areas near the equator do not experience drastic changes in temperature throughout the year, limiting the utility of the detection method, based on the change of the trend. To avoid possible issues, records of *H. rufipes* in a band of 10º northern and southern to the equatorial line were removed.

The remaining variables were built according to these temperature-dependent seasons. For temperature, calculations include (a) the annual and seasonal amplitude (calculated for each season), (b) the percentiles 10, 25, 50, 75 and 90 of the complete daily series, (c) the accumulated degree days annually and for each season, (d) the accumulated degree days above 0 ºC annually and for each season, and (e) the slope of spring-summer and summer-autumn (that explains how fast the spring turns into summer, and how quickly the temperature falls from summer to autumn). For actual evapotranspiration (AET), soil humidity (SOIL) and water vapor pressure deficit in the atmosphere (VPD), the same variables were calculated both annually and for each season, including the accumulated ones, except for the values recorded below 0 ºC.

### Further selection of the explanatory variables

Before building the definition of the niches of either *H. marginatum* or *H. rufipes*, it was necessary to remove the variables that lack enough variation among the *three sets of points*, as well as the pairs of variables that are correlated between them and that are redundant, overfitting the definition of the niche. For example, the accumulated temperatures above and below 0 ºC in a period of time are negatively correlated; it is necessary to remove one of them because both have the same ecological meaning. An ANOVA allowed to retain the variables with highest meaning among the three datasets. The variables with a *p* value > 0.05 were removed from the dataset as they do not contribute enough to separate the environmental niche of the three species (i.e., the two *Hyalomma* spp. and the ‘absence dataset’). On the other hand, the absolute values of pair-wise correlations between any two variables detect correlated ones, using the R package *caret* (Kuhn 2008). If two variables have a high correlation (threshold set at the absolute value of 0.6), the one with the highest absolute correlation with other variables is removed. After such preliminary filtering a total of 41 variables remained. The calculation of an n-dimensional hypervolume, involves the description of the overlap of *n* variables. This was done in the Orange programming environment, a set of Python programming resources freely available from https://orangedatamining.com.

### Testing the ability of the selected variables to define the niche

Models were used to test whether the most significant variables were selected in the previous step and whether the definition of the niche for each species was accurate enough. They were based on algorithms of regression and classification that operate on numerical data to obtain the probability of a response (presence/absence). The algorithms selected were (i) Random Forest, (ii) Gradient Boosting, and (iii) AdaBoost. As mentioned, models were not intended to produce predictive maps, but to demonstrate that the variables selected were the most discriminatory of the niches of the two *Hyalomma* spp. in comparison with the ‘negative dataset’. As a proof of concept, accumulated variables were entered as raw values and also as the effective value above a threshold of 0 ºC. It has been reported that activity of ticks begins above a given threshold (e.g., Hancock et al. 2011). These empirical observations are not questioned in this study, even if they are contrary to laboratory findings (Tomkins et al. 2014) or if they do not correlate with findings on tick metabolic activity (Alasmari and Wall 2021).

**Fig. 2:**
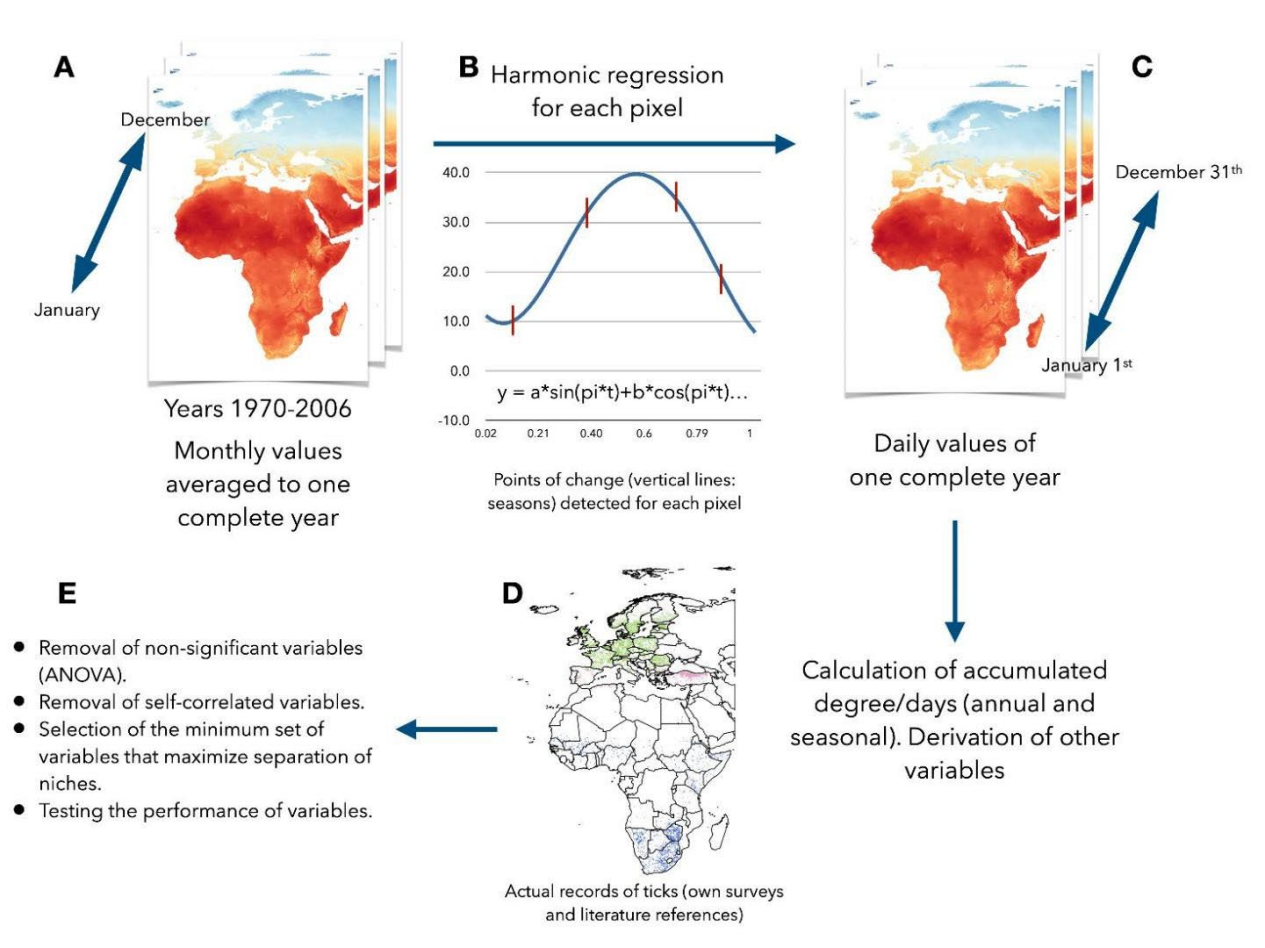
The flow of work in the current study, using monthly data of the Terraclimate repository (**A**) for the years 1970–2006, for the variables average temperature, soil humidity, atmospheric vapor pressure deficit, and actual evapotranspiration. (**A**) Harmonic regression (**B**) produced the daily data (**C**) from the average monthly values of each variable; other variables were calculated for the complete year and temperature-derived seasons. Annual and seasonal accumulated temperature were calculated from these daily values. Each point of the recorded distribution of the ticks (both positive and negative values) was ‘loaded’ with the whole set of values of each variable (**D**). Final steps (**E**) involved cleaning of the dataset of climate data and tick collections, including (1) the removal of non-significant variables (i.e., not discriminant among datasets), (2) removal of the correlated variables, and (3) the testing of the performance of the remaining variables separating the points of the datasets by several modeling algorithms

Machine learning models must be evaluated to determine their effectiveness, based on true positives (TP), true negatives (TN), false positives (FP) and false negatives (FN). For all the algorithms above, several measures were calculated. The *Classification Accuracy* is simply the measure of how many observations the model correctly predicted over the total number of observations: (TP + TN)/(TP + TN + FP + FN). *Precision* is the measure of how many observations of the model correctly predicted over the number of correct and incorrect predictions: TP/(TP + FP). *Recall* is the measure of how many observations the model correctly predicted over the total amount of observations: TP/(TP + FN). The *F1 Score*, maximizes both *precision* and *recall* scores for a model. All the definitions before were obtained from the online documentation of the Orange programming environment, available at https://orange3.readthedocs.io/projects/orange-data-mining-library/en/latest/reference/evaluation.cd.html (accessed June 2022). If the previous steps correctly selected the minimum number of best explanatory variables, the algorithms should separate the niche of each tick species with almost 100% reliability. Low values of predictive values would point to a wrong selection of the explanatory variables. The reliability of the models was measured by the ‘area under the curve’ (AUC) (Jiménez-Valverde 2012). The flow of work for this study is included in Fig. 2.

## Results

The complete set of climate data for each record in the three datasets, is available in Supplementary Material 1. Several variables produced a good discrimination among the three datasets (Fig. 2). Other than traits representing the complete year, climate data observed for some seasons were most representative of the presence/absence of each tick species. Most discriminant variables include accumulated temperature in the complete year and some seasons, as well as the sum and amplitude of VPD in winter and the complete year. Other variables produced a low decrease in AUC and were ignored in further analyses. To note, every variable regarding water in the soil and evapotranspiration were removed from the combination that better defines the niches.

**Fig. 3:**
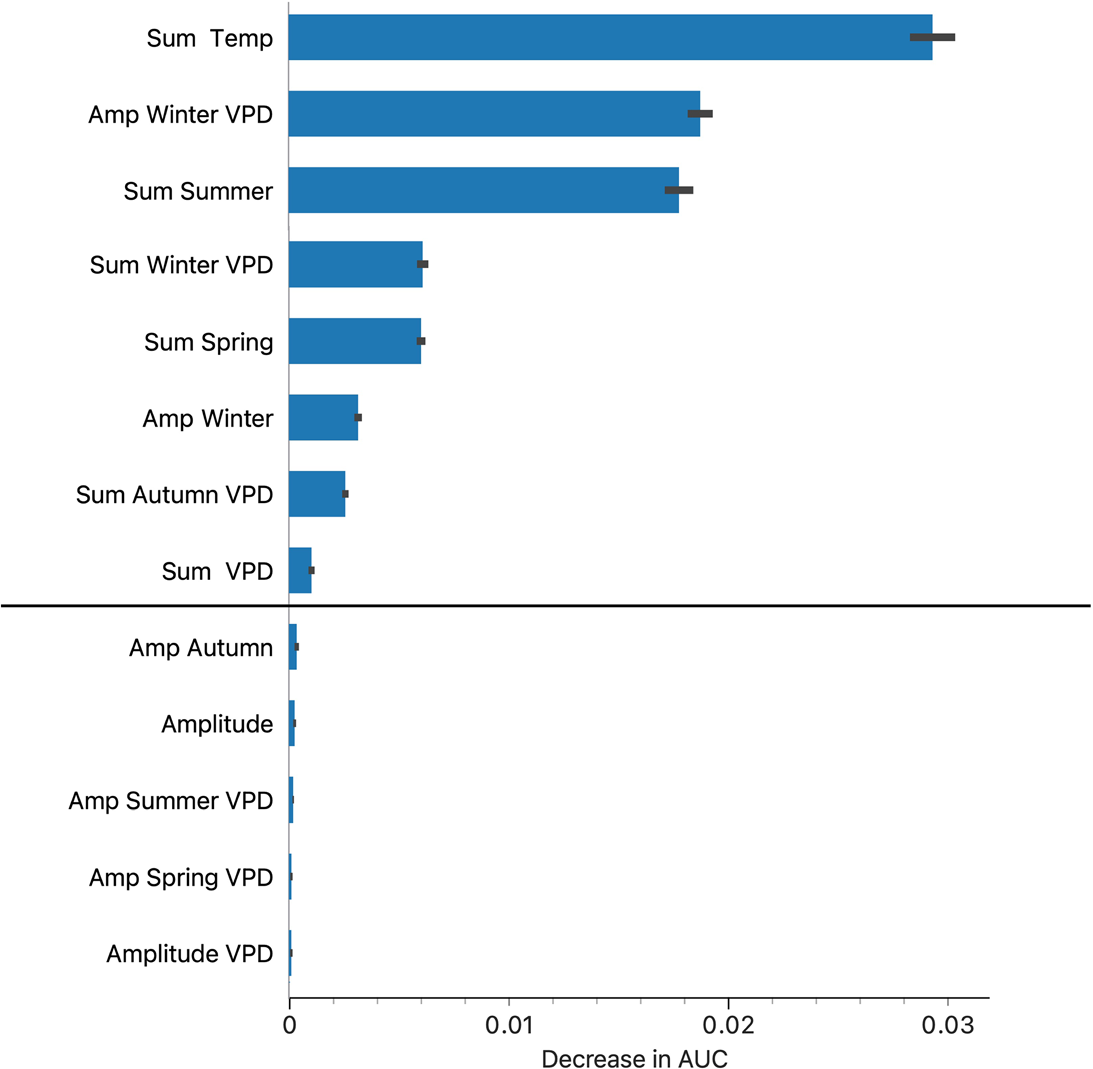
The most significant variables separating the climate niches between both *Hyalomma marginatum* and *H. rufipes*, and these from the negative dataset (points of collections of *Ixodes ricinus*). From top to bottom, annual sum of temperature, amplitude of vapor pressure deficit (VPD) in winter, sum of temperature in summer, sum of VPD in winter, sum of temperature in spring, amplitude of temperature in winter, sum of VPD in autumn, and annual sum of VPD. Variables below the straight line are secondary to the abiotic niche definition, including amplitude of temperature in autumn, annual amplitude of temperature, amplitude of VPD in summer, amplitude of VPD in spring, and annual amplitude of VPD. Bars represent average values in the decrease in the values of the area under the curve (AUC) used as a measure of the importance of each variable. Such a set of variables was issued without the use of accumulated temperature above the threshold of 0 ºC

Variables related to the water in the air performed better in all tests (together with temperature) than variables related to humidity of soil. Of interest, too, no variables related with absolute maximum or minimum values of any variables were selected as candidates for such niche definition: the two *Hyalomma* spp. cannot be separated between them or with the group of negative records using any combination of ‘extreme’ variables (absolute maxima or minima). Table 1 displays the evaluation of every model in the determination of the niche for each species, using the variables in Fig. 3.

**Table 1:** Evaluation of the reliability of three algorithms in the separation of the climate niche of two species of *Hyalomma* ticks and the negative set based on presences of *Ixodes ricinus*, using the set of variables included in Fig. 3. The definitions for Classification Accuracy (CA), Precision, Recall and F1 Score are included in the Material and Methods section

Species	Model	CA	Precision	Recall	F1 Score
*H. marginatum*	Random Forest	0.982	0.960	0.930	0.940
	Gradient Boosting	0.992	0.980	0.970	0.970
	AdaBoost	0.987	0.960	0.960	0.960
* H. rufipes*	Random Forest	0.996	0.995	0.980	0.988
	Gradient Boosting	0.999	0.999	0.998	0.999
	AdaBoost	0.999	0.998	0.997	0.998
*I. ricinus*	Random Forest	0.984	0.984	0.993	0.988
	Gradient Boosting	0.992	0.992	0.996	0.994
	AdaBoost	0.987	0.989	0.992	0.990

**Fig. 4:**
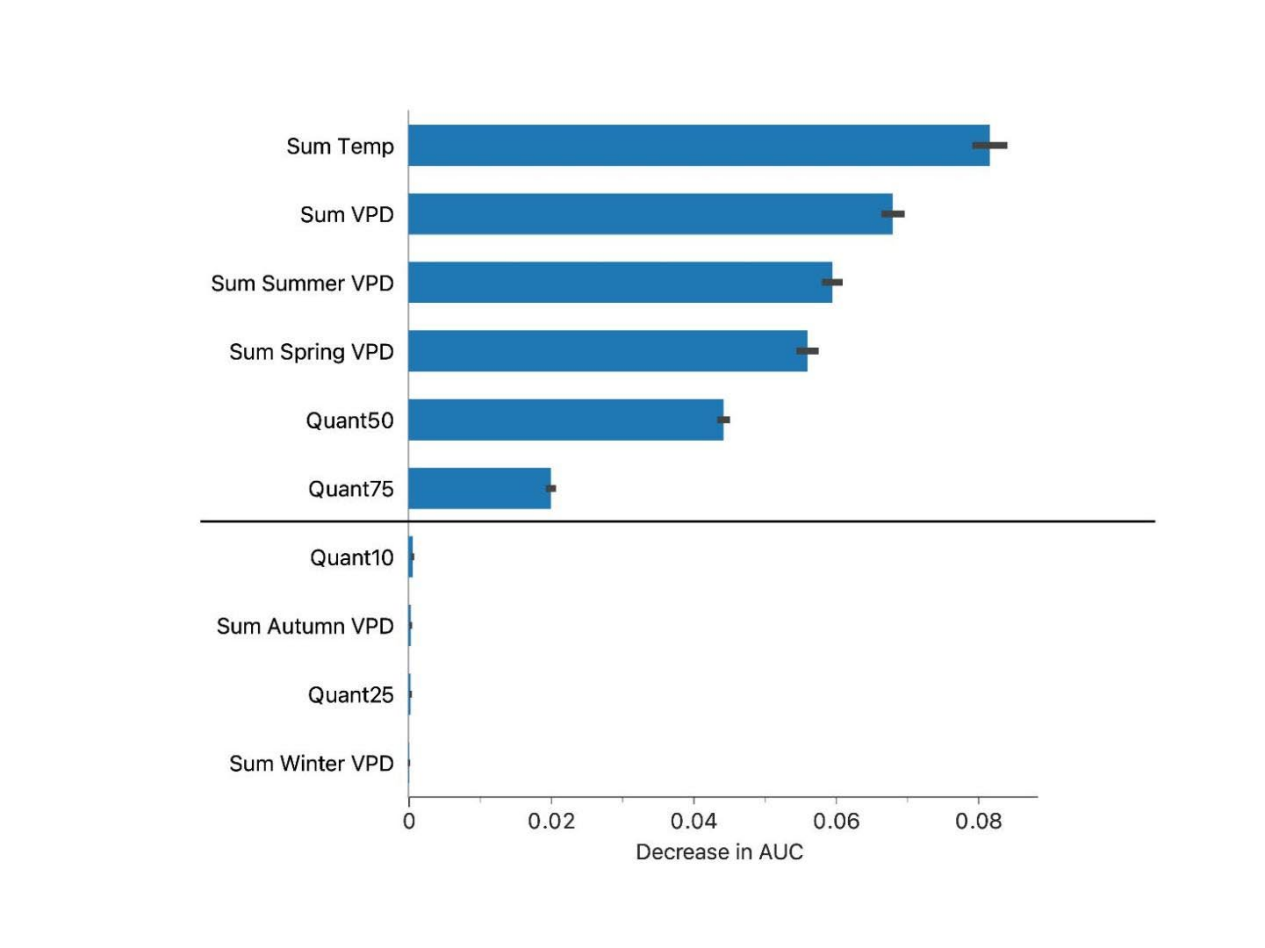
The most significant variables separating the climate niches between both *Hyalomma marginatum* and *H. rufipes*, and these from the negative dataset (points of collections of *Ixodes ricinus*) forcing the removal of seasonal accumulated temperature and allowing inclusion of annual and seasonal accumulated temperature above the threshold of 0 ºC (which were not selected as discriminatory variable). This set of variables performed ca. 40% poorer in the discrimination of the abiotic niches than the set of variables displayed in Fig. 3. From top to bottom, annual sum of temperature, annual sum of temperature, sum of vapor pressure deficit (VPD) in summer, sum of VPD in spring, quantile 50 and 75 of temperature. Variables below the straight line are secondary to the abiotic niche definition, including quantile 10 of temperature, sum of VPD in autumn, quantile 25 of temperature, and sum of VPD in winter. Bars represent average values in the decrease in the values of the area under the curve (AUC) used as a measure of the importance of each variable. Such a set of variables was issued without the use of accumulated temperature above the threshold of 0 ºC

The forced inclusion of the temperature values above a threshold resulted in the selection of a different set of variables. Six variables were selected as the most representative delineating the niches of the target ticks, as shown in Fig. 4. However, when these variables were used to train the classification algorithms, all the evaluation tests dropped significantly implying a drastic decrease of the classificatory power of about 40% (Table 2).

**Table 2:** Evaluation of the reliability of three algorithms in the separation of the climate niche of two species of *Hyalomma* ticks and the negative set based on presences of *Ixodes ricinus* using the set of variables included in Fig. 4, resulting from the forced inclusion of accumulated temperature above a threshold. The definitions for Classification Accuracy (CA), Precision, Recall and F1 Score are included in the Material and Methods section

Species	Model	CA	Precision	Recall	F1 Score
*H. marginatum*	Random Forest	0.504	0.611	0.601	0.608
	Gradient Boosting	0.602	0.667	0.670	0.670
	AdaBoost	0.690	0.750	0.721	0.740
* H. rufipes*	Random Forest	0.510	0.540	0.520	0.520
	Gradient Boosting	0.550	0.590	0.580	0.580
	AdaBoost	0.490	0.510	0.470	0.490
*I. ricinus*	Random Forest	0.450	0.491	0.456	0.474
	Gradient Boosting	0.510	0.550	0.563	0.558
	AdaBoost	0.435	0.419	0.437	0.428

**Fig. 5:**
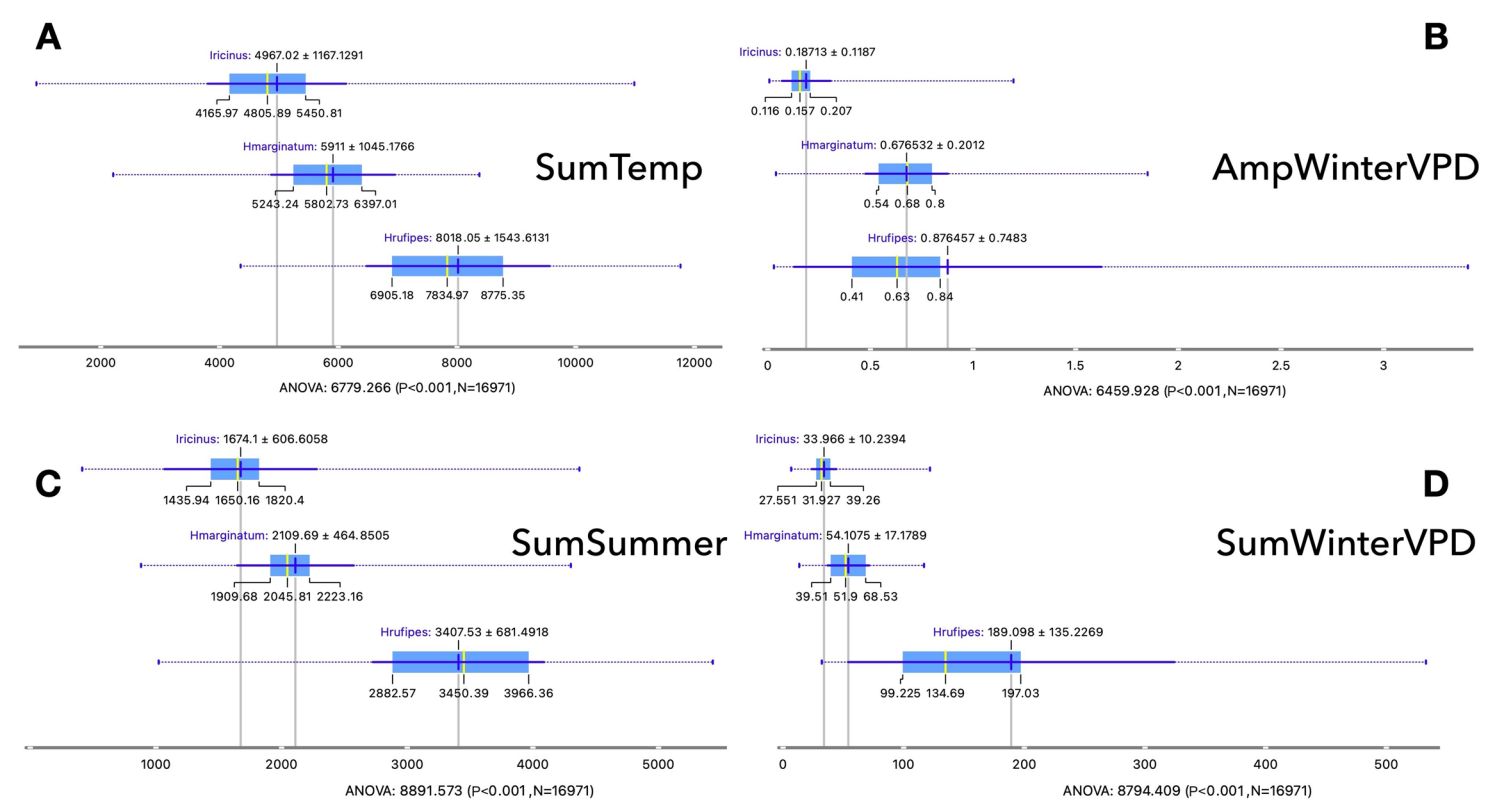

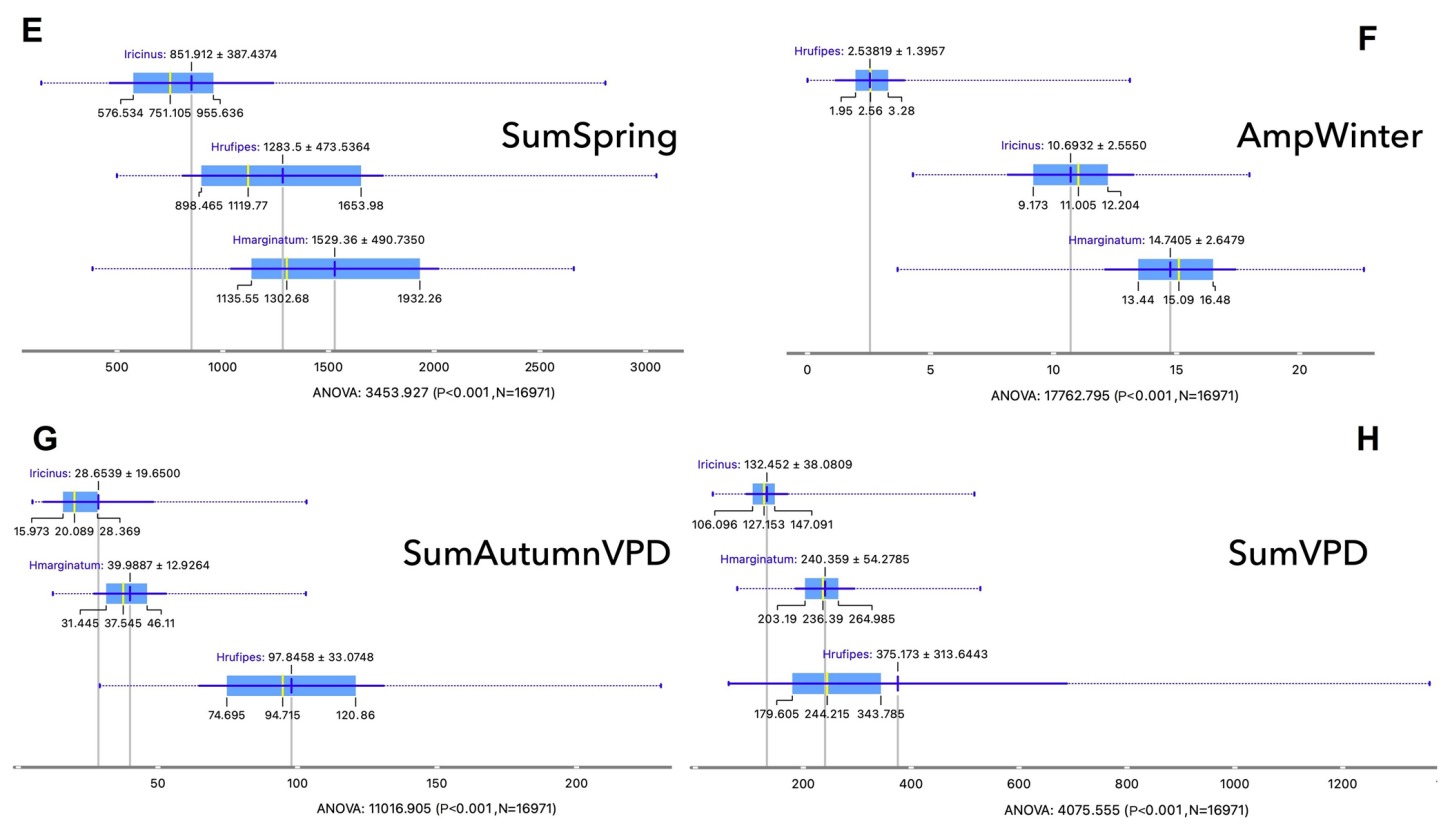
Box and whiskers displaying the range of values of the most significant variables defining the abiotic niche of *Hyalomma marginatum* and *H. rufipes*, in comparison with the negative dataset (presence of *Ixodes ricinus*). Each chart shows the range of values for each species, as the mean, the median, and the percentiles 10, 25, 75, and 100. Included are (**A**) the annual sum of temperature, (**B**) the amplitude of vapor pressure deficit (VPD) in winter, (**C**) the sum of temperature in summer, (**D**) the sum of VPD in winter, (**E**) the sum of temperature in spring, (**F**) the amplitude of temperature in winter, (**G**) the sum of VPD in autumn, and (**H**) the annual sum of VPD.

The ranges of values of the best discriminatory variables that explain the niches of either *H. marginatum* or *H. rufipes* are described in Fig. 5 (shown in the same order of importance detected by the Rank filter in Fig. 3). Note that the ranges of some variables may overlap for different tick species, but the focus is the joint use of these variables to describe the niche. For example, the annual accumulated temperature shows a difference of almost 2,000 degree-days for the niche of both *Hyalomma* spp. (Fig. 5A) or > 1,000 ºC warmer in comparison with the negative dataset. The amplitude of temperature in winter is clearly higher for both *Hyalomma* spp., covering a range between 9.7 ºC (percentile 25 of *H. marginatum*) and 16.5 ºC (percentile 75 of *H. rufipes*; Fig. 5F); the amplitude of temperature in winter for the negative sites varies between 1.95 and 3.3 ºC. This is an important finding, because it points to the high thermal tolerance of both *Hyalomma* spp. to a large range of temperature variability in winter. *Hyalomma rufipes* always prefers warmer sites, mainly in summer (3,400 ± 681 vs. 2,100 ± 464 ºC for *H. marginatum*; Fig. 5G). However, differences are only 140 ºC of accumulated temperature in spring between both *Hyalomma* spp. (450–650ºC compared against the negative set). Both *H. marginatum* and *H. rufipes* do not show clear preferences for very dry habitats, but they favor sites with a large amplitude of values of VPD. Both *Hyalomma* spp. prefer sites between 240 and 375 kPa of VPD accumulated throughout the year.

**Fig. 6:**
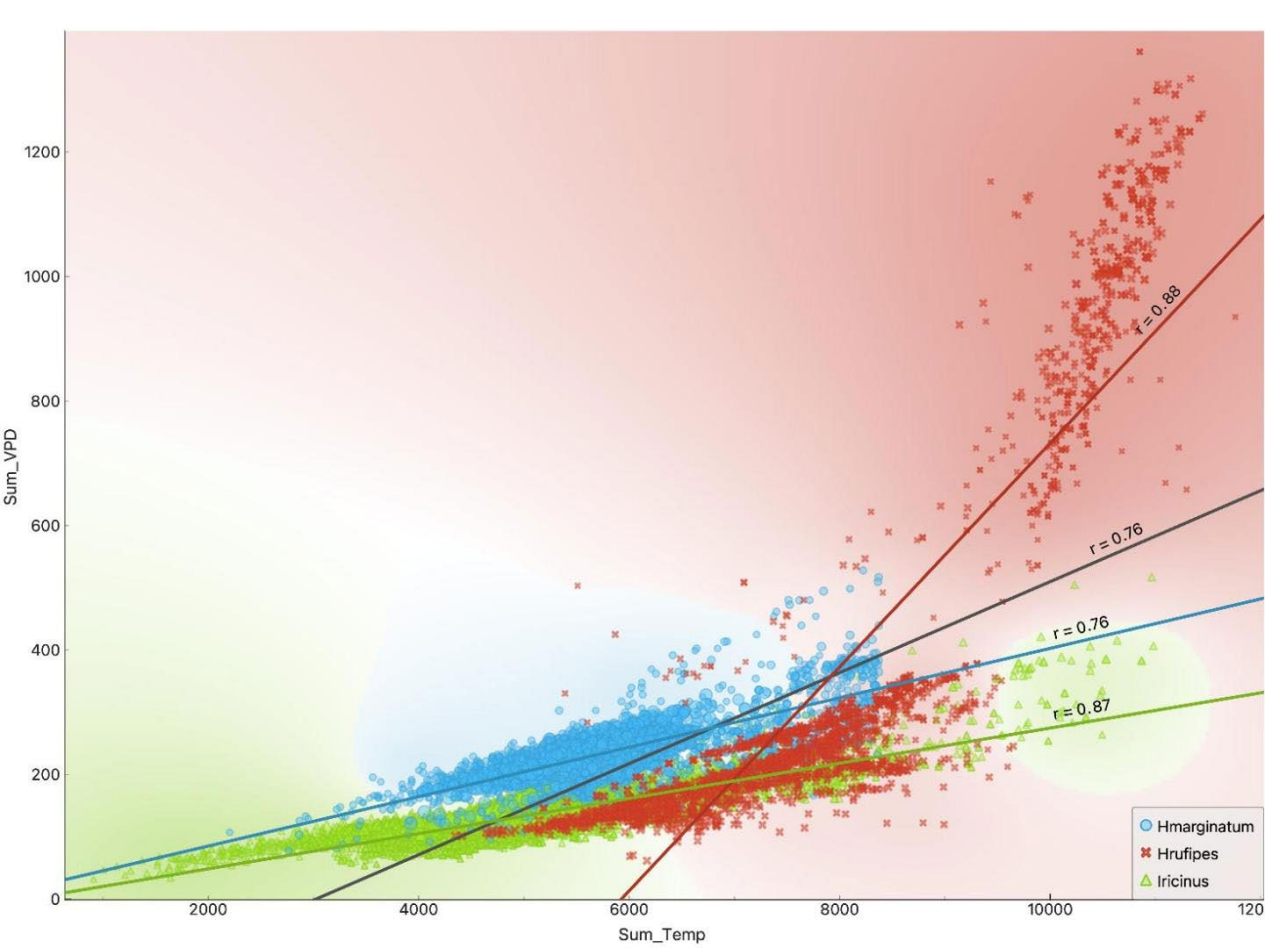
Separation of the niches of *Hyalomma marginatum* and *H. rufipes* and the negative dataset (presence of *Ixodes ricinus*) using the values of the annual sum of temperature and the annual sum of vapor pressure deficit (VPD). Colors in the background follow the same colors used for displaying the points of each species and mean for the separation to the optimal set of values for each species

**Fig. 7:**
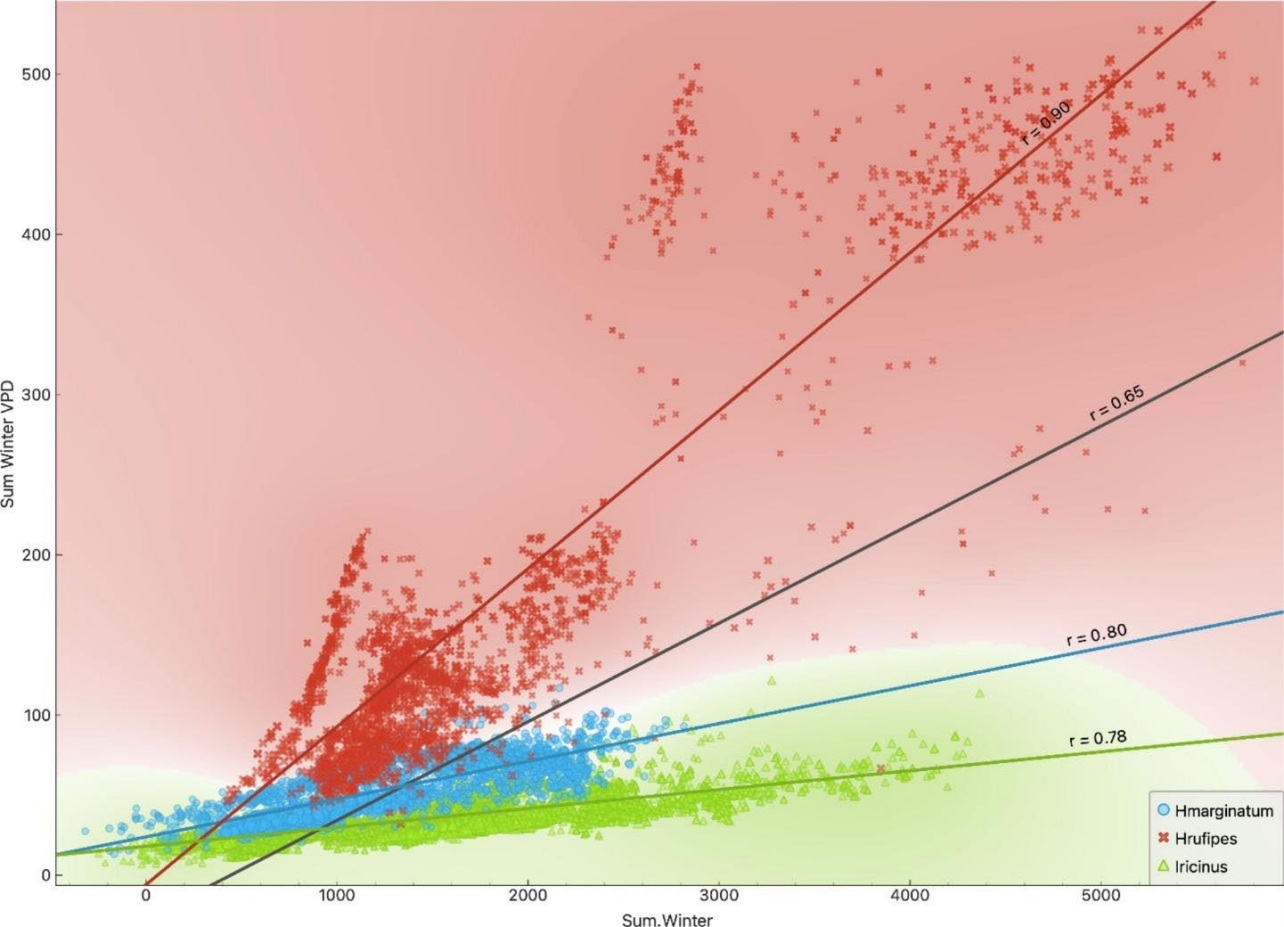
Separation of the niches of *Hyalomma marginatum* and *H. rufipes* and negative dataset (presence of *Ixodes ricinus*) using the values of the sum of temperature in winter and the sum of vapor pressure deficit (VPD) in winter. Colors in the background follow the same colors used for displaying the points of each species and mean for the separation to the optimal set of values for each species

The framework allows the explicit comparison of the range of variables defining the niche of both species of *Hyalomma* in comparison with the dataset of *I. ricinus*. Figure 6 shows the delineation of the niche for the datasets of occurrence of the three ticks using only the accumulated annual temperature and the accumulated annual VPD. *Hyalomma marginatum* cannot exist in areas under 3,000–4,000 ºC per year (there is only one record of the tick below such range of accumulated temperature). For *H. rufipes*, this limit is about 4,500 ºC at rates of annual VPD around 150 kPa. However, a group of sites positive for *H. rufipes* has high values of both accumulated temperature and VPD, which matches the previous comments about the preferences towards high amplitude of values of these traits. In contrast, the negative set (or the sites where *I. ricinus* exists) is associated to an accumulated annual temperature of only 1,000 ºC at very low VPD. This is indicative of slow development together with low mortality because of high values of water in the atmosphere near the ground. Regarding the winter conditions that favor the presence of *Hyalomma* spp. (Fig. 7) it results that both *H. marginatum* and *I. ricinus* support similar lower limits of winter accumulated temperature. The finding that some sites with permanent populations of *I. ricinus* are up to 1,400 ºC warmer than those supporting permanent populations of *H. marginatum* supports these comments. To note, this finding derives from the calculation of the seasonality. At northern latitudes in Europe, the winter lasts more days than in the Mediterranean; therefore, a longer winter allows a higher accumulation of temperature.

Results indicate that the accumulated temperature alone (annual or seasonal) is not enough to characterize the niches that could be occupied by the alleged invasive ticks. *Hyalomma marginatum* prefers sites of higher VPD (or, roughly translated, higher water deficit). Of interest, no permanent populations of *H. marginatum* have been reported under an annual accumulated temperature of 3,000–3,400 ºC. The upper limit of temperature at which the species has been found is consistently located around 8,000 ºC. It is necessary to note that both sets of variables (temperature and atmospheric water-related) act jointly in the delineation of positive/negative sites for the two alleged invasive *Hyalomma* spp., and that VPD has an actual impact on the presence/absence of the tick. The use of one single set of variables (i.e., temperature) should not be considered as the only restricting trait modulating the distribution of the ticks targeted in this study. In other words, cold winters should not be considered the hallmark for the absence of *Hyalomma* ticks.

## Discussion

This study addressed the description of the environmental niches of *H. marginatum* and *H. rufipes*, two invasive species of ticks in Europe, for which the capacity of permanent colonization of new territories is currently unknown. This study focused on both the finding of a minimal set of descriptive variables for each species retaining an ecological meaning, as opposite to the option based on the reduction to a set of principal components. As such, the climate niche lacks traits derived from hosts abundance, the effects of human disturbance, landscape features and fragmentation, or vegetation. The analysis is focused on climate with a progressive filtering of attributes, selecting the variables producing the best delineation of niches. This study aimed to demonstrate the value of simple procedures to adequately select the variables that unambiguously define the gradient of variables promoting lasting tick populations. The most important findings are (i) the combined effect of accumulated temperature and VPD in the definition of these niches, (ii) the lack of importance of evapotranspiration and soil humidity, (iii) the lack of resolutive power of accumulated temperature above a threshold, and (iv) the absence of absolute maximum or minimum values in the definition of such niches.

The last decade has seen an increase of reports of either *H. marginatum* or *H. rufipes* in Central and Northern Europe, and the British Isles (Chitimia-Dobler et al. 2016; Hansford et al. 2019; Hubálek et al. 2020; Rudolf et al. 2021; Földvári et al. 2022; Lesiczka et al. 2022). The number of reports cannot automatically translate into an ‘increased abundance’ but are perhaps due to an ‘increased awareness’ together with a probable increased survival (or molting) of the ticks in the invaded sites. Nevertheless, the recording of *Hyalomma* spp. northern to their historical range in the Mediterranean basin or Africa, respectively, is a common finding. Morel (1965) already mentioned adult ticks of *H. marginatum* in Finland, Denmark, Germany, Sweden, and the UK; Aeschlimann and Büttiker (1975) reported *H. marginatum* in Switzerland. The issue is *if*, *where*, or *when* in the coming years, the climate would turn suitable for the populations of these imported ticks allowing completion of the life cycle. Relatively many adults of *H. marginatum* have been reported so far in southern France (Vial et al. 2016), the rest of records in the target territory being only isolated adults. This seems to be a consequence of the molt and survival of a few engorged nymphs, resulting in few, geographically isolated adults, that could not meet and mate (Vial et al. 2016).

Statistical comparisons need a set of sites in which the target ticks have not been recorded. Similar studies use a set commonly called ‘pseudo-absences’, which are random sites selected by an algorithm where the target organism has not been reported. This is probably a procedure not fully reliable, because pseudo-absences are difficult to select to avoid overlapping with the realized distribution. As the choice of pseudo-absence points is especially problematic in species with high sampling bias or gathered without adequate sampling designs (Descombes et al. 2022) the approach in the current study has been the selection of a set of presences of other tick species, in this case *I. ricinus*. The choice is not casual, because it is probably the best surveyed tick in Europe. Points positive to *I. ricinus* mean that these sites have been surveyed for ticks; we can guess that, if present, *Hyalomma* spp. would have been reported because of their novelty in the region. There is no complete ‘presence-absence’ correlation among datasets because *Ixodes* and *Hyalomma* ticks are collected by different methods, probably not comparable among them, but this procedure seems to be a good conciliation for a reliable set of negative sites. In any case, this is one of the limitations of the current study. Others could be derived from the lack of adequate sampling of *Hyalomma* spp. out of its historical range. To note, only the ticks found on the ground by either active or passive surveys may have a reliable geo-reference. However, those collected on birds, even performing short-distance travels, cannot be mapped adequately, and the inferred climate could be unreliable.

Extreme care in exploring the adequate variables was also observed, removing the non-discriminatory or self-correlated ones, selecting the smallest set of variables with ecological meaning that can correctly separate the niches of the target species. Aim was to demonstrate that a careful selection (not algorithmically driven) of variables is very informative. Daily climate data are essential for the definition of the climate niche of ticks. Most of the datasets available online lack a daily time resolution, or miss some important data (like relative humidity, water vapor pressure, or any other measure of water in the air) that are known to affect the tick’s life cycle (Alonso-Carné et al. 2015). However, methods based on time series analysis allow the decomposition of monthly data into daily ones (Estrada-Peña et al. 2014) enabling the calculation of accumulated values. Anyway, such extra statistical processing of the variables may be another limitation of this study that could derive into an unwanted variability of the raw climate data, that could be easily replaced by modern satellite-derived images. Data on precipitation were purposely rejected because they have a low impact on the tick’s life cycle (Hubálek et al. 2003; Berger et al. 2014; Kiewra et al. 2014) and probably act only on local scales or under special characteristics of vegetation or soil. Rainfall and relative humidity are poorly correlated, at least in the biogeographical domains of Europe (Alonso-Carné et al. 2015).

The probability of completion of the life cycle of *Hyalomma* spp. in Europe (incl. the British Isles) has been commonly addressed using an approach based only on the accumulated temperature, probably misinterpreting a previous study on the modelling of the life cycle of *H. marginatum* (Estrada-Peña et al. 2011) indicating that the tick could not complete its life cycle below 3,000 degree-days. This figure matches the results presented in this study, validating the current statistical approach, against results obtained from laboratory colonies. Results also confirmed that VPD is a fundamental descriptor of the niche of the target ticks. The fact that accumulated VPD in autumn and winter are involved as two of the most important climate variables discriminating positive and negative sites, indicated the importance of water air contents in cold periods for *Hyalomma* ticks. It is noteworthy that accumulated temperature over a threshold (annual or seasonal) was never selected as candidate for niche definition. It has been repeatedly reported that ticks enter a state of torpor below a critical threshold of temperature, stopping questing if temperature drops below a species-specific threshold – something that has been criticized on empirical data (Tomkins et al. 2014) or does not match laboratory data on metabolic rates (Alasmari and Wall 2021). The findings of the current study support these previous reports. As these studies demonstrated that the response to a temperature threshold is a genetic trait, such a value should not be used for studies on large areas, because it is most probably a hallmark of the regional population of a species.

Permanent populations of *H. marginatum* or *H. rufipes* seem to be controlled by the joint action of the water in the air and the accumulated temperature: the medium–high values of water deficit detected in sites where the tick exists would control the mortality, whereas the accumulated temperature would regulate the development (i.e., molt, oviposition, and incubation). We cannot discard local adaptations to prevailing climate conditions. It has been reported that relative humidity is decreasing over mid latitudes in Europe in a trend along a period of about 10 years leading up to 2008 (Simmons et al. 2010). A lower relative humidity is inversely proportional to higher values of VPD in the atmosphere, which corresponds with an increase of the territory suitable in the parts of central Europe. Such verified decrease of relative humidity together with the trend to warmer conditions over wide areas of Europe could be responsible of the recent findings of adult *Hyalomma* in areas out of its historical range of distribution. The need of a minimum accumulated temperature to complete the development processes still holds; nevertheless, this study shows that the use of such threshold *alone* to evaluate probabilities of persisting populations may be too simplistic. Conclusions include the recommendation of exploring different sets of climate data, whose importance as explanatory variables is easily verifiable by simple statistical methods, considering that the study of the metabolic rates of ticks may be fertile field of research to elaborate around a new modeling paradigm.

## Electronic supplementary material

Below is the link to the electronic supplementary material.

Supplementary Material 1

## Data Availability

The datasets generated during and/or analyzed during the current study are available as supplementary material in this manuscript.
